# Hard X-ray omnidirectional differential phase and dark-field imaging

**DOI:** 10.1073/pnas.2022319118

**Published:** 2021-02-22

**Authors:** Hongchang Wang, Kawal Sawhney

**Affiliations:** ^a^Diamond Light Source Ltd., Harwell Science and Innovation Campus, Didcot, OX11 0DE, United Kingdom

**Keywords:** X-ray phase contrast, dark field, material science, X-ray speckle

## Abstract

Ever since the discovery of X-rays, tremendous efforts have been made to develop new imaging techniques for unlocking the hidden secrets of our world and enriching our understanding of it. X-ray differential phase contrast imaging, which measures the gradient of a sample’s phase shift, can reveal more detail in a weakly absorbing sample than conventional absorption contrast. However, normally only the gradient’s component in two mutually orthogonal directions is measurable. In this article, omnidirectional differential phase images, which record the gradient of phase shifts in all directions of the imaging plane, are efficiently generated by scanning an easily obtainable, randomly structured modulator along a spiral path. The retrieved amplitude and main orientation images for differential phase yield more information than the existing imaging methods. Importantly, the omnidirectional dark-field images can be simultaneously extracted to study strongly ordered scattering structures. The proposed method can open up new possibilities for studying a wide range of complicated samples composed of both heavy, strongly scattering atoms and light, weakly scattering atoms.

## Significance

We demonstrate an approach to generate a new type of X-ray imaging mode, which is called omnidirectional X-ray differential phase imaging. The proposed method enables us to detect the subtle phase changes in all directions of the imaging plane, which complements conventional X-ray imaging methods with information that they cannot provide. Importantly, the omnidirectional dark-field images can also be simultaneously retrieved for studying a wide range of complicated samples, particularly strongly ordered systems. The extracted information will not only provide insights into the microarchitecture of materials, but also enrich our understanding the macroscopic behavior. The presented technique could potentially open up numerous practical imaging applications in both biomedical research and materials science.

Over the past two decades, various X-ray directional differential phase contrast and dark-field imaging techniques have been developed for applications in nondestructive testing, biomedical imaging, and materials science (1–6). The X-ray differential phase is directly linked to the local gradient of the sample's phase shift. Since the phase shift term is much larger than the absorption term for materials composed of light elements, X-ray differential phase imaging offers high contrast at a lower radiation dose than conventional absorption contrast imaging requires. In addition, X-ray dark-field images describe the scattering power due to the structural variation and density fluctuation. Importantly, directional X-ray dark-field imaging detects the orientation of anisotropic structures at micrometer scale and provides valuable information about the scattering power of samples (7–10). Sometimes, directional dark-field imaging can fail to pick up the scattering of some weakly absorbing samples, while X-ray differential phase contrast imaging can show these weak signals more clearly. Therefore, these two techniques can provide complementary information to each other.

However, directional differential phase contrast and dark-field imaging can measure only the component of electron density changes and small-angle scattering in the scanning direction. As a result, the risk of overlooking structures with strong but varying orientation within the sample is high (11, 12). Therefore, it will be desirable to generate the omnidirectional phase shift changes or dark-field signals to pick up detailed information along all directions. To retrieve the omnidirectional dark-field signals, advanced phase gratings and absorption masks have been proposed and tested on anisotropic microstructures (13–16), but the need for such special optics, along with stringent experimental setup and poor spatial resolution, has restricted its application. In principle, the omnidirectional differential phase images could be retrieved by scanning the optics in various directions or rotating the samples (17, 18), but this would increase the complexity of the experimental setup and dramatically prolong the data acquisition time.

Recently, the speckle-based imaging technique has been developed and become popular for its great simplicity and remarkable performance (19, 20). The horizontal and vertical differential phase information can be retrieved from either a single speckle image or a stack of speckle images, depending on whether speed or spatial resolution is more important. In addition, the directional dark-field image can be retrieved from the one-dimensional (1D) speckle scanning technique (10), while two separated scans are required to obtain the orthogonal dark-field images.

In this study, we describe an algorithm to extract both the omnidirectional differential phase and dark-field signal with a randomly structured wavefront modulator, such as a sandpaper. To achieve the omnidirectional information and minimize the necessary radiation dose, the sandpaper is scanned along a spiral trajectory. Fourier analysis is then performed to obtain the phase changes and scattering signal of the sample in all directions of the imaging plane. In addition, the proposed method shows great potential to decouple the isotropic and anisotropic scattering signals by analyzing the omnidirectional dark-field images.

## Method and Data Processing

A speckle pattern (in the x,y plane) can be generated by inserting a randomly structured wavefront modulator into the beam. Several speckle-based methods for collecting high-resolution phase contrast and dark-field images have been investigated by scanning the modulator in different directions (21–25). For the two-dimensional (2D) speckle scanning technique, the modulator was scanned along horizontal (h) and vertical (v) directions with constant step size smaller than the speckle size (21). The virtual speckle $\left(I_{h,v}\right)$ was built from the collected intensity signal during the two transverse scans. Even though the differential phase and dark-field images along both horizontal and vertical directions can be simultaneously provided by the 2D speckle scanning technique, the collection and processing of hundreds of images is time-consuming.

To avoid the 2D raster scan, speckle vector tracking (SVT) and unified modulated pattern analysis (UMPA) methods have been proposed independently by scanning the modulator with nonuniform step size (22, 25). Both the horizontal and vertical differential phase contrast images and dark-field images can be generated from the UMPA and SVT methods. However, neither method can provide the directional dark-field information since the speckle data analysis is carried out within a subset window $\left(I_{x,y}\right)$ by applying a cross-correlation algorithm with a different merit function (26).

The 1D speckle scanning approach has been developed to provide directional dark-field image by scanning the modulator along one direction (10, 23). The virtual speckle image ($I_{h,y}$ for horizontal scan) is generated from the scanning direction (h) and the orthogonal speckle window direction (y). Consequently, the sensitivity of the two orthogonal differential phase images is dependent on the scanning step size and detector pixel size. Nevertheless, the modulator still has to be scanned along the horizontal and vertical directions separately in order to obtain two directional dark-field images.

To circumvent the above limitations, we propose an alternative approach to analyze the speckle data and achieve differential phase and dark-field images in all directions. In order to reduce the image numbers and data acquisition time, the spiral scan method is used for high-speed ptychography experiments (27). Similarly, in our work, we scan the modulator in a spiral trajectory (u). The number of images for a spiral scan are similar to the one for a 1D scanning method. Importantly, the reduced number of images can also significantly reduce the data processing time compared to the 2D scanning method.

**Fig. 1.**
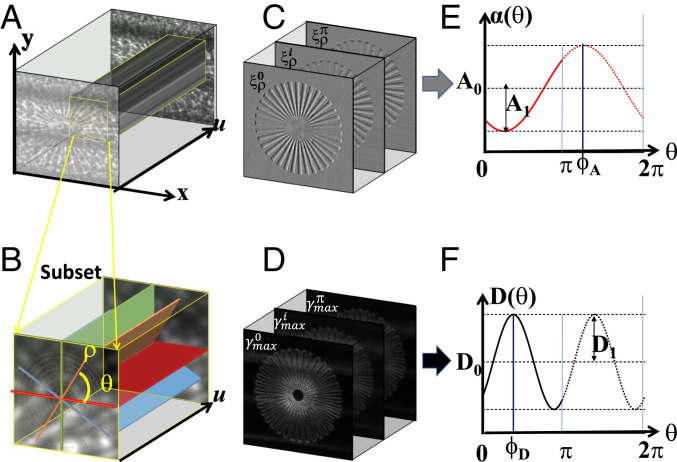
A schematic illustration of the extraction of the directional differential phase and dark-field images of a sample star pattern. (*A*) The collected stack of speckle images in Cartesian coordinates. (*B*) The select subset from *A* is transformed into polar coordinates. (*C* and *D*) The extracted speckle displacement ${\text{ξ}}_{\rho }^{\text{θ}}$ along the polar directions and the maximum of the cross-correlation coefficient $\gamma_{max}^{\text{θ}}$ images. (*E* and *F*) The differential phase and dark-field signal modulation as the polar angle changes from 0 to 2π.

One stack of reference speckle images $F\left(x,y,u\right)$ was first collected without sample in the beam by performing the spiral scan, and then the same scan was repeated to generate the sample speckle image stack $G\left(x,y,u\right)$ when the sample was moved into the beam. As illustrated in Fig. 1*A*, for each pixel (m,n) in the speckle image plane (x,y), a surrounding subset with window size $W_{S}\times W_{S}$ pixels was selected. In order to build the virtual speckle images in polar coordinate, the same procedure as described in previous work was followed (14). The local Cartesian coordinate (x,y) was transformed into the polar coordinate (ρ,θ) with number of θ values *M* by 2D interpolation and expressed as

$$x=\rho \text{cos}\theta +x_{0},\;y=\rho \text{sin}\theta +y_{0}.$$ [1]

Here, $\left(x_{0},y_{0}\right)$ is the origin of the local Cartesian coordinate, and the angle θ varies from 0 to *π*. The corresponding transformed virtual speckle stack in polar coordinate is shown in Fig. 1*B*. Therefore, the virtual sample speckle image $g_{\rho ,u}^{\theta }$ can be built by combining the speckle image along the polar direction *ρ* and the spiral scan direction u, and the same procedure can be repeated for the reference (no sample) speckle image $f_{\rho ,u}^{\theta }$ with slightly larger window size $W_{R}\times W_{R}$ $\left(W_{R}=W_{S}+2E_{W}\right)$ for meaningful cross-correlation operation. Here, the edge size $E_{W}$ defines the searching range for cross-correlation algorithm, and it should be chosen large enough to track the maximum speckle displacement for given experimental parameters. In addition, the minimum window size $W_{S}$ for the sample speckle image is set to 3 in order to convert into polar coordinates and obtain the directional information. Unlike the circular grating with fixed unit-cell periodicity [**5**], the window size for the proposed method can be freely chosen based on the requirement of spatial resolution and sample properties. For the optimization of speckle scanning parameters, the methodology is very similar to the conventional SVT and UMPA technique. The scan range is usually much larger than the average speckle size so that the virtual speckle intensity can be varied randomly along the scan direction. In addition, the improved angular sensitivity can be achieved by increasing the number *N* of speckle images per scan, while the single-image speckle-tracing method is a special case when *N* is equal to 1. Further description about the speckle data analysis can be found in *SI Appendix*.

The correlation coefficient maps $\gamma_{\rho ,u}^{\theta }$ were calculated by cross-correlating the two virtual speckle images (21):

$$\gamma_{\rho ,u}^{\theta }=f_{\rho ,u}^{\theta }⋆g_{\rho ,u}^{\theta }=\sum \sum \frac{\left(f_{\rho ,u}^{\theta }-\bar{f_{\rho ,u}^{\theta }}\right)\times \left(g_{\rho ,u}^{\theta }-\bar{g_{\rho ,u}^{\theta }}\right)}{\Delta f_{\rho ,u}^{\theta }\times \Delta g_{\rho ,u}^{\theta }}.$$ [2]

Cross-correlation is represented by the pentagram symbol ⋆, and $\bar{f_{\rho ,u}^{\theta }}$ $\left(\bar{g_{\rho ,u}^{\theta }}\right)$ and $\Delta f_{\rho ,u}^{\theta }$ $\left(\Delta g_{\rho ,u}^{\theta }\right)$ are the mean and SD of the subset, respectively. Once the cross-correlation coefficient maps $\gamma_{\rho ,u}^{\theta }$ are calculated, the speckle displacement is corresponding to the maximum value $\gamma_{max}^{\text{θ}}$. Although integer pixel displacements can be easily calculated, the integer pixel resolution is far from the accuracy requirement for deriving differential phase. Both tracking accuracy and computation efficiency are the key parameters for subpixel registration algorithm. Here, the correlation coefficient curve-fitting method is chosen as subpixel registration algorithm for its simplicity and high efficiency. Therefore, the speckle displacement $\xi_{\rho }^{\theta }$ along the polar direction and $\xi_{u}^{\theta }$ along the scan direction can be precisely calculated from the coordinate of the maximum of the cross-correlation coefficient $\gamma_{max}^{\text{θ}}$ (28).

The 1D speckle scanning technique can be treated as a special case (θ=0 or π/2) of the proposed method. Note that in this treatment, two factors affect the maximum of the cross-correlation coefficient: the small-angle X-ray scattering, and the second derivative of the phase due to the abrupt change at the sample edges. The dark-field signal $D^{\theta }$ is defined as the second moment of the scattering angle distribution, and $\alpha^{\theta }$ is the directional differential phase (23, 29). They can be derived from the above pixelwise data analysis and expressed as$\{\begin{array}{c} \alpha^{\theta }=\frac{2\pi P}{\lambda L}\xi_{\rho }^{\theta }\\ D^{\theta }=\frac{-{\bar{\varsigma }}^{2}}{8\pi^{4}L^{2}}\text{ln}\gamma_{max}^{\theta } \end{array},$ **[3]**

where P and $\bar{\varsigma }$ are the detector’s pixel size and the average speckle size, respectively. L represents the distances between the sample and the detector, and λ is the wavelength. For simplicity, the pixel subscripts x,y are not explicitly added to $D^{\theta }$, $\alpha^{\theta }$ and other quantities defined from here onward in this study.

The directional dark-field images do not distinguish between scattering at orientation angles θ and θ+π. In contrast, the phase gradient will change from positive to negative (or vice versa) when the orientation angle changes from θ to θ+π, and the period of the differential phase images is 2π. However, differential phase images $\alpha^{\theta }$ can be derived from the above data processing only on the range θ=[0,π]. Here, we generate a stack of differential phase images $A^{\theta }=\left[\alpha^{\theta },-\alpha^{\theta }\right]$ by combining the existing stack $\alpha^{\theta }$ with its duplicated and negative stack $-\alpha^{\theta }=\alpha^{\theta +\pi }$. Then, the fast Fourier transform (FFT) analysis can be applied to $A^{\theta }$, and the directional differential phase images can sense the orientation of phase changes from 0 to 2*π* in the imaging plane. Following the definition similar to previous works (7, 13), the cosine model was used to extract the average and amplitude of scattering and differential phase and the corresponding main orientation. It can be expressed as follows:

$$\{\begin{array}{c} A^{\theta }=A_{0}-A_{1}\text{cos}\left(\theta +\phi_{A}\right)\\ D^{\theta }=D_{0}-D_{1}\text{cos}\left(2\theta +\phi_{D}\right) \end{array}.$$ [4]

Here, the Fourier coefficients $A_{0}\left(D_{0}\right)$ and $A_{1}\left(D_{1}\right)$are related to the average and amplitude differential phase (dark field), and the phase term $\phi_{A}$ and $\phi_{D}$ is the main orientation of the differential phase and dark field, respectively. The Fourier coefficients and the phase term for each pixel can then be extracted by an FFT analysis. The differential phase averaged over θ, $A_{0}$, should be zero because by definition $\alpha^{\theta }+\alpha^{\theta +\pi }=0$.

The horizontal and vertical differential phase images $\left(\alpha^{x}\;\text{and}\;\alpha^{y}\right)$ can be calculated from the amplitude $\left(A_{1}\right)$ and main orientation $\left(\phi_{A}\right)$ of the differential phase image from Eq. **4**. by setting θ to 0 or π/2. It can be written as

$$\{\begin{array}{c} \alpha^{x}=-A_{1}\text{cos}\left(\phi_{A}\right)\\ \alpha^{y}=A_{1}\text{sin}\left(\phi_{A}\right) \end{array}.$$ [5]

Furthermore, the phase shift *Φ* induced by the sample can then be reconstructed from the two transverse phase gradients $\left(\alpha^{x}\;\text{and}\;\alpha^{y}\right)$ by using the Fourier transform integration method and can be written as in ref. (17):

$$\Phi \left(x,y\right)={ℱ}^{-1}\left[\frac{ℱ\left[\alpha^{x}+i\alpha^{y})\right]\left(k,l\right)}{2\pi i\left(k+il\right)}\right]\left(x,y\right),$$ [6]

where ${ℱ}^{-1}\left(ℱ\right)$ is the inverse (forward) Fourier operations and (k,l) represent the reciprocal-space coordinates corresponding to (x,y). The expression of $\alpha^{x}\;\text{and}\;\alpha^{y}$ from Eq. **5** was then substituted into Eq. **6**. According to Euler’s formula, the phase shift Φ can be expressed with

$$\Phi \left(x,y\right)={ℱ}^{-1}\left[\frac{ℱ\left[-A_{1}e^{-i\phi_{A}}\right]\left(k,l\right)}{2\pi i\left(k+il\right)}\right]\left(x,y\right).$$ [7]

Hence, the phase shift Φ can be directly calculated from the derived amplitude $A_{1}$ and main orientation $\phi_{A}$ of the directional differential phase images.

### Experiment 1.

The principle of the proposed technique was validated by experimental measurements at the Diamond Light Source’s B16 Test beamline (30). X-rays with an energy of 15 keV were selected from the bending magnet source using a double-multilayer monochromator. As shown in Fig. 1*A*, a piece of sandpaper with a grain size of 5 µm was chosen as a modulator, which was mounted on a 2D piezostage and located 45 m from the X-ray source. The sample was fixed on a motorized stage 375 mm downstream of the modulator. The distance between the sample and detector was L = 955 mm. Images of the speckle pattern were collected using a high-resolution X-ray camera composed of a pco.edge charge-coupled device (CCD) detector and a microscope objective with a LuAG (Ce) scintillator (31). As a demonstration of the capabilities of the proposed technique, a phantom with 36 actinomorphic star patterns (QRM) was purposefully chosen because its features are distributed along all the directions in the imaging plane. In addition, a woodlouse sample was also tested because it has complex biological structures containing both isotropic and anisotropic scattering properties.

To image the star-pattern phantom, the camera system was focused with an effective pixel resolution of 0.5 μm × 0.5 μm. The sandpaper was scanned with a spiral trajectory over a range of 100 µm, with *n* = 50 images recorded. Two stacks of speckle images were taken, one without and one with the sample in the X-ray beam. The exposure time per speckle image was 2 s. Fig. 1*A* shows the stack of speckle images with the sample present. The selected window is then transformed in polar coordinate and is shown in Fig. 1*B*. The virtual speckle stack is then generated along the polar direction *ρ* and the spiral scan direction u. Following the above procedure, as shown in Fig. 1 *C* and *D*, both the differential phase and the dark-field images can be obtained from the same dataset by applying the pixelwise cross-correlation algorithm by following the above procedure. As demonstrated in Fig. 1 *E* and *F*, the Fourier coefficients ($A_{0},$ $A_{1},$ $D_{0}$, and $D_{1}$) and the phase term $\phi_{A}$ and $\phi_{D}$ can then be extracted by using the FFT analysis for differential phase and dark-field image stacks. In order to successfully apply the FFT analysis to the directional data, the number of rotation angles M should be equal to or larger than 3. In our case, $E_{S}=5$, $W_{S}=3$, M=8, and N=50 were used to improve the spatial resolution. Further discussion about the parameter’s optimization can be found in *SI Appendix*.

Data processing was performed using a program run in MATLAB version 2020b. A Gaussian filter was applied to the raw speckle images to minimize the speckle noise and improve the tracking accuracy. The MATLAB code was run using a standard desktop computer (Dell with Intel Xeon 3.70-GHz processor and 64-GB memory), and the data processing is equally distributed over eight workers with Parallel Computing Toolbox. It took 26 min for processing the phantom with region of interest 950 × 950 pixels by using the above parameters.

**Fig. 2.**
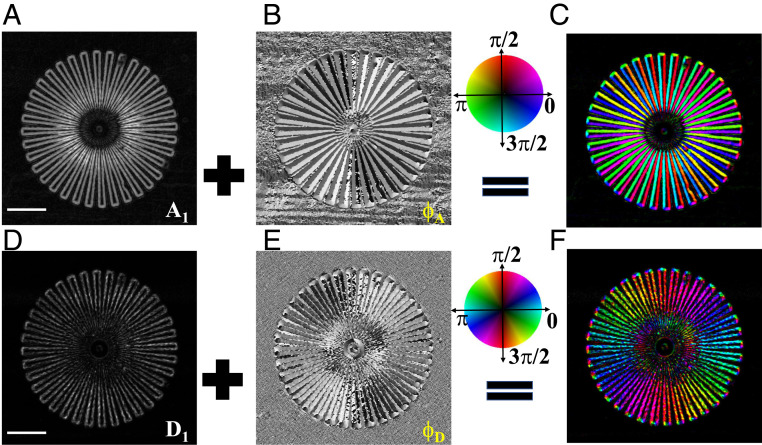
(*A* and *B*) The retrieved amplitude $A_{1}$ and main orientation $\phi_{A}$ of differential phase. (*C*) The constructed omnidirectional differential phase, as rendered in an HSV color scheme. (*D* and *E*) The corresponding retrieved amplitude $D_{1}$ and main orientation $\phi_{D}$ of dark field. (*F*) The constructed omnidirectional dark field, as rendered in an HSV color scheme. The gray color for *A* and *D* indicates the range of normalized amplitude $A_{1}$ and $D_{1}$ from 0 (dark) to 1 (bright). For *B* and *E*, the gray shades cover the range from 0 (dark) to 2*π* (bright). (Scale bar, 0.2mm.)

Fig. 2 shows the retrieved differential phase amplitude $A_{1}$ and dark-field amplitude $D_{1}$ and the corresponding main orientation $\phi_{A}$ and $\phi_{D}$. Unlike the conventional unidirectional dark field and differential phase, the amplitude of the omnidirectional dark field and differential phase remains uniform around the outer edge of the star. Since the star patterns are etched silicon, the sensitivity of the differential phase is much higher than the corresponding scattering signal. Both the amplitude and the main orientation for the differential phase image show better contrast than the ones for the dark-field image. In order to show the directional information, the hue-saturation-value (HSV) color scheme is used with the hue given by the orientation ($\phi_{A}$ and $\phi_{D}$), the value equal to the corresponding amplitude ($A_{1}$ and $D_{1}$), and the saturation equal to 1 (7, 13). Fig. 2 *C* and *F* are the directional differential phase and dark field rendered in an HSV color scheme. It should be noted that the color for the directional dark field is the same for angle θ and θ+π, while it is different for the directional differential phase (15). Therefore, the directional differential phase not only identifies the orientation of the phase change, but also its sign. Here, only the edge of the star patterns can be observed from the directional dark-field image, whereas the directional differential phase image also shows the direction of the phase gradient on each bar.

### Experiment 2.

**Fig. 3.**
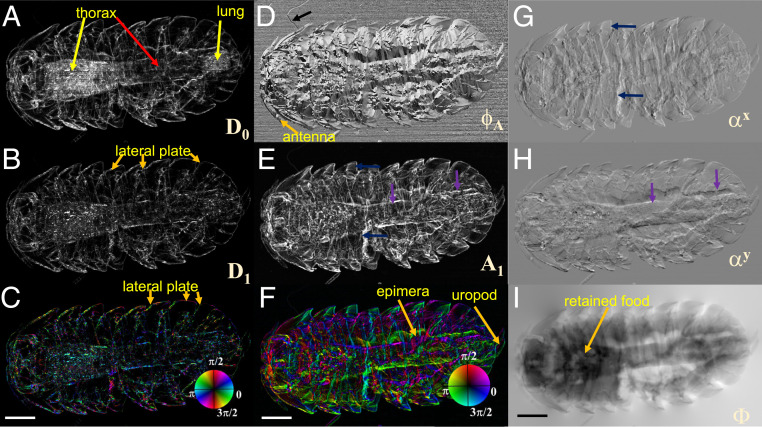
(*A* and *B*) The retrieved average $D_{0}$ and amplitude $D_{1}$ of dark-field signal of a woodlouse sample. The gray color indicates that the normalized $D_{0}$ and $D_{1}$ changes from 0 (dark) to 1 (bright). (*C*) The constructed omnidirectional dark field, as rendered in an HSV color scheme. (*D* and *E*) The main orientation $\phi_{A}\left[0,\;2\pi \right]$ and normalized amplitude $A_{1}\left[0,\;1\right]$ of differential phase. (*F*) The constructed omnidirectional differential phase, as rendered in an HSV color scheme. (*G* and *H*) The calculated horizontal and vertical differential phase from $A_{1}$ and $\phi_{A}$. The gradient varies from −3 to 3 μrad (bright). (*I*) The reconstructed phase from *G* and *H*, in which the phase ranges from −60 (dark) to 60 rad (bright). (Scale bar, 0.5mm.)

A woodlouse was then selected to demonstrate the applicability of the proposed method for the study of biological samples with complicated structures. The specimen was clamped inside a plastic film box so that the X-rays can pass through the sample with minimum scattering and phase distortion from the container. The effective pixel resolution of the imaging system was increased to 3.9 μm × 3.9 μm to ensure the entire sample was within the field of view, and the exposure time was reduced to 500 ms per frame. The differential phase and phase images are presented in Fig. 3. The woodlouse exhibits a few distinctive features in various image modes. The same speckle scanning and processing parameters and procedures as the star phantom were used for the woodlouse sample. As shown in Fig. 3 *A* and *B* the higher scattering signals for the edge of the lateral plate are shown in both the average $\left(D_{0}\right)$ and amplitude $\left(D_{1}\right)$ of the directional dark-field images for the woodlouse. On the other hand, the lung and part of the thorax (marked with arrows in Fig. 3*A*) show strong average scattering but only very weak directional scattering. This indicates that the lung tissue and the retained food (containing rotting plants) in the thorax show isotropic scattering signals. The average scattering map shows both isotropic and anisotropic scattering signals, while the directional scattering map shows only the anisotropic scattering signals. The anisotropic scattering signal will change with the detecting direction of the dark-field image, while the isotropic scattering signal will scatter light evenly in all directions. Therefore one can decouple the isotropic and anisotropic scattering signals of the woodlouse’s microstructure by comparing the average and amplitude scattering maps. In the HSV colormap of the directional scattering signal in Fig. 3*C*, the directional scattering of the lateral plane can be clearly identified along different orientations. As mentioned above, the second derivative of the phase signals will also affect the derived dark-field signals. Therefore, the edges of the antenna and thorax can all be seen and coded with directional information. The corresponding differential phase images from Fig. 3*D* to Fig. 3*E* show complementary information to the dark-field images. It should be noted that Fig. 3*D* is the main orientation for differential phase, and the orientation angle is independent of the sample’s phase shift (thickness). A small hairlike structure (marked with black arrow) is clearly seen in Fig. 3*D*, while it is hardly visible in all the other image modes. Consequently, the main orientation angle image for differential phase can provide enhanced contrast to the weak phase signal, and it can be used to study multiple features of complex samples. The phase changes along the epimera and uropod can be spotted from the constructed differential phase image with HSV colormap, but are not visible in the directional dark image. Since the directional differential phase image displays the orientation, it can be used to separate different orientation structures for interpretive visualization. For example, we can show or hide the features with horizontal, vertical, or any other orientation angles.

The calculated horizontal and vertical differential phase images are shown in Fig. 3 *G* and *H*. The vertical features along the lateral plate are clearly visible in the horizontal differential phase image $\left(\alpha^{x}\right)$, while they are hardly noticeable in the vertical differential phase image $\left(\alpha^{y}\right)$, and vice versa. In contrast, all the horizontal and vertical features marked in Fig. 3 *G* and *H* can be simultaneously shown in the amplitude of the differential phase image (in Fig. 3*E*), which provides the phase changes along all directions. Therefore, the directional differential phase image gives insight into the orientation of the phase changes in the sample. It should be pointed out that these two images $\left(\alpha^{0}\;and\;\alpha^{\frac{\pi }{2}}\right)$ can be directly extracted from the raw speckle images, but only $\rho_{s}\times N$pixels along horizontal and vertical directions are used for speckle data analysis. In contrast, the angular sensitivity of calculated $\alpha^{x}\;\text{and}\;\alpha^{y}$ images is somewhat higher since they are averaged over the speckle window $\rho_{s}\times \rho_{s}\times N$.

As shown in Fig. 3*I*, the phase image provides a comprehensive overview of the internal structure of the woodlouse. For example, the retained foods from the thorax are clearly visible in both the reconstructed phase map (Fig. 3*I*) and the corresponding average dark-field image (Fig. 3*A*). By contrast, the soft tissues such as the uropod and epimera are barely visible in the dark-field images, yet can be clearly distinguished from the surrounding lateral plate in the omnidirectional differential phase image (Fig. 3*F*).

## Discussion and Conclusion

In summary, we have demonstrated that the omnidirectional differential phase and dark-field images can be simultaneously extracted from a single dataset with the use of a simple modulator. We have shown that the retrieved multimodal images from the proposed approach can reveal a wide variety of internal structures within one sample. The directional differential phase images reveal the directional dependence of the weakly absorbed features, complementing the directional dark-field images. In addition, the retrieved main orientation image of the directional differential phase can further improve the contrast for thin samples, while the amplitude image of the directional differential phase shows the phase changes along all directions in the imaging plane at once. Moreover, high-quality horizontal and vertical differential phase images and phase shifts can be automatically calculated from the above amplitude and main orientation images. Unlike in the previously reported 1D dark-field imaging technique, the isotropic and anisotropic scattering signals from underlying microstructure can be potentially decoupled by applying Fourier analysis to the omnidirectional dark-field images. The omnidirectional differential phase and dark-field images resolve the directional dependence of complex microstructures, which is inaccessible to conventional X-ray imaging techniques.

Compared to the other techniques, high-precision optics is not required and rotation of the samples or the optics can be avoided. Only a piece of sandpaper is required as a wavefront modulator to generate the speckle pattern. In addition to the low cost, robustness, and availability in large sizes, sandpaper with different grain sizes can be chosen to suit the spatial resolution requirement of the detector. In addition, the modulator can be replaced with a strongly absorbing mask for the study of thick, dense materials with high-energy X-rays (32, 33). The spatial correlation length can be adjusted to study variable feature sizes by choosing a suitable window size. It should be noted that it is not essential to scan the modulator with a spiral trajectory for the proposed method. In fact, it can be scanned with either a random step or a constant step along the transverse directions. The data collected from the SVT and UMPA can be used to produce omnidirectional differential phase images and dark-field images by the proposed algorithm. The experimental parameters can also be optimized according to the requirements on speed or sensitivity. Although the proposed technique has been demonstrated with synchrotron radiation, it can be potentially transferred to laboratory X-ray microfocus sources for wider application (23, 34, 35). The radiation dose can be further reduced by using a highly efficient flat-panel detector. Finally, the proposed omnidirectional method paves the way for X-ray scattering tensor tomography for the inspection of biomedical and material science samples (16, 36).

## Supplementary Material

Supplementary File

## Data Availability

All study data are included in the article and/or *SI Appendix*.
