# Supplementary material for: Hard X-ray omnidirectional differential phase and dark-field imaging
Source: Proc Natl Acad Sci U S A. 2021 Feb 22;118(9):e2022319118. doi: 10.1073/pnas.2022319118 (PMC7936267; doi:10.1073/pnas.2022319118)
Supplement: Supplementary File [file pnas.2022319118.sapp.pdf]

PNAS
www.pnas.org

Supplementary Information for

Hard X-ray omnidirectional differential phase and dark-field imaging

Hongchang Wang and Kawal Sawhney

Corresponding Author: Hongchang Wang

Email: hongchang.wang@diamond.ac.uk

This PDF file includes:

Supplementary text
Figs. S1 to S3
SI References

Support Information Text

The stack of sample (and reference) speckle images $G(x,y,u)$ (and $F(x,y,u)$) are collected with (and without) sample in the beam by performing the spiral scan. As illustrated in Fig. S1 (c), for each pixel (m,n) in the sample (and reference) speckle image plane (x,y), a surrounding subset with window size $W_S \times W_S$ (and $W_R \times W_R$) pixels are selected. For the conventional SVT and UMPA technique(1, 2), the cross correlation is performed between the reference subset image $f_{x,y}$ and sample subset image $g_{x,y}$ after the sample is inserted into the beam. The Pearson correlation coefficient is used to track the speckle displacement.

In contrast, as illustrated in Fig. S1 (c), the local Cartesian coordinate (x,y) for each subset image was transformed into the polar coordinate (ρ,θ) with number of θ values for the proposed technique. Therefore, the virtual sample (and reference) speckle subset image $g^{\theta}_{\rho,u}$ (and $f^{\theta}_{\rho,u}$) are built by combining the speckle image along the polar direction ρ and the spiral scan direction u. For clarity, only the virtual speckle subset image at angle $\theta = \pi/3$ is shown. This generated virtual speckle subset $f^{\pi/3}_{\rho,u}$ is shown in Fig. S1 (a) with the window size $W_R \times N_R$ pixels. In order to perform the cross-correlation algorithm, the sample speckle subset should be smaller than the reference speckle subset. Therefore, the window size for the sample speckle subset is $W_S \times N_S$ in Fig. S1 (b). Here, $E_W = (W_S - W_R)/2$ and $E_N = (N_S - N_R)/2$ is the edge size along the polar direction and spiral scan direction, respectively. Consequently, the correlation coefficient map $\gamma^{\pi/3}_{\rho,u}$ for the pixel (m,n) is calculated by cross-correlating the two virtual speckle images $f^{\pi/3}_{\rho,u}$ and $g^{\pi/3}_{\rho,u}$. The effective size of the correlation coefficient maps is $(W_S - W_R + 1) \times (N_S - N_R + 1)$ pixels. The calculated cross-correlation coefficient maps $\gamma^{\pi/3}_{\rho,u}$ is shown in Fig. S1 (d), and the peak coordinate and amplitude can be precisely calculated with the correlation coefficient curve-fitting method with sub-pixel accuracy. The speckle displacement $\xi^{\pi/3}_{\rho}$ along the polar direction and the peak amplitude $\gamma^{\pi/3}_{max}$ is marked in the circular dot in Fig. S1 (e) and (f) , which are generated after performing the pixel-wise analysis following the above procedure. The directional differential phase and dark-field images at angle $\theta = \pi/3$ is calculated. As described in the manuscript, the number of θ values M was used for the polar coordinate transformation. The same data analysis process is then repeated for different θ values to achieve the multiple directional differential phase and dark-field images, which are shown in Fig.1 (c) and (d).

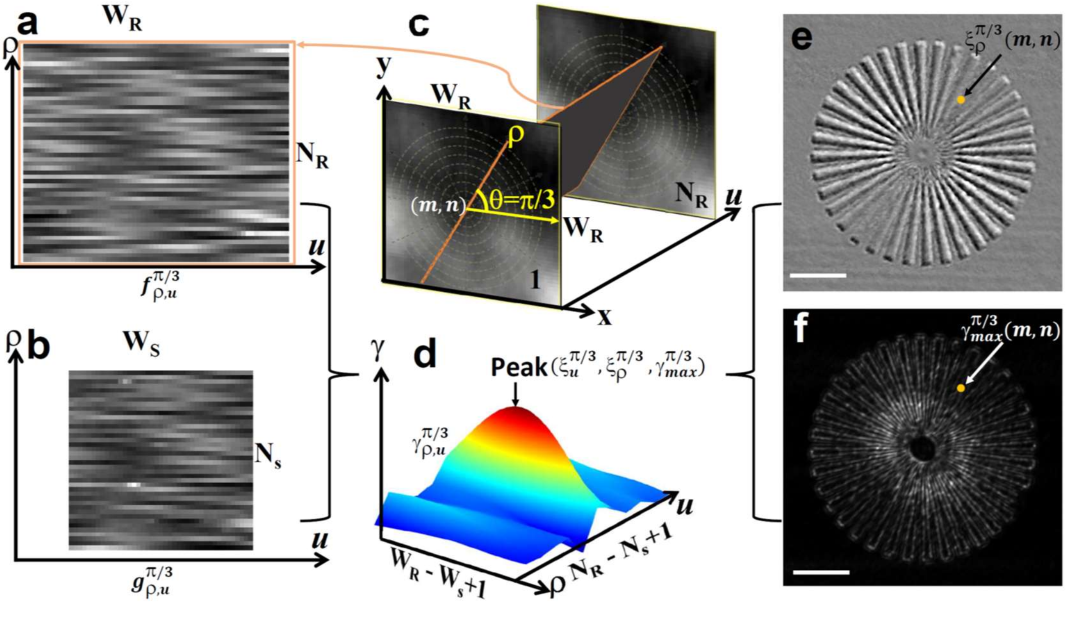

Fig. S1. A schematic illustration of the data processing for the differential phase and dark-field images at angle $\theta = \pi/3$ of a sample star pattern. (a) and (b) the generated virtual reference and sample speckle image after the polar coordinate transformation from Cartesian, (c) A schematic illustration for transformation from Cartesian coordinate (x,y) into the polar coordinate (ρ,θ) for pixel (m,n). (d) The calculated cross-correlation coefficient map from virtual reference and sample speckle image (a) and (b). (e) and (f) the extracted speckle displacement $\xi^{\pi/3}_{\rho}$ along the polar directions and the maximum of the cross-correlation coefficient $\gamma^{\pi/3}_{max}$ images.

For the optimization of data acquisition and processing parameters, the methodology of the proposed speckle imaging technique is very similar to the SVT, UMPA, and 1D speckle scanning technique(1-3). The angular sensitivity is one of the important parameters that determines the quality of a differential phse image. One commonly used procedure to verify the angular sensitivity is to calculate the standard deviation of the wavefront gradient in empty space (4, 5). Following this approach, we have tracked the speckle displacement between two stacks of reference speckle images to quantify angular sensitivity. As shown in Fig.S2, the horizontal and vertical angular sensitivity has been calculated by changing the window size W_S and number of speckle images N. As expected, the angular sensitivity of the differential phase will be improved by increasing the number of speckle images N and the window size W_S. Although better angular sensitivity of the differential phase image can be achieved with more speckle images, it comes at the price of longer data

acquisition and data processing time. Moreover, with more speckle images, one also increases the radiation dose, which is crucial for the study of biological samples and medical applications. Hence, a trade-off has to be made to choose the optimal number of speckle images that achieves moderate angular sensitivity without excessive radiation doses or data processing.

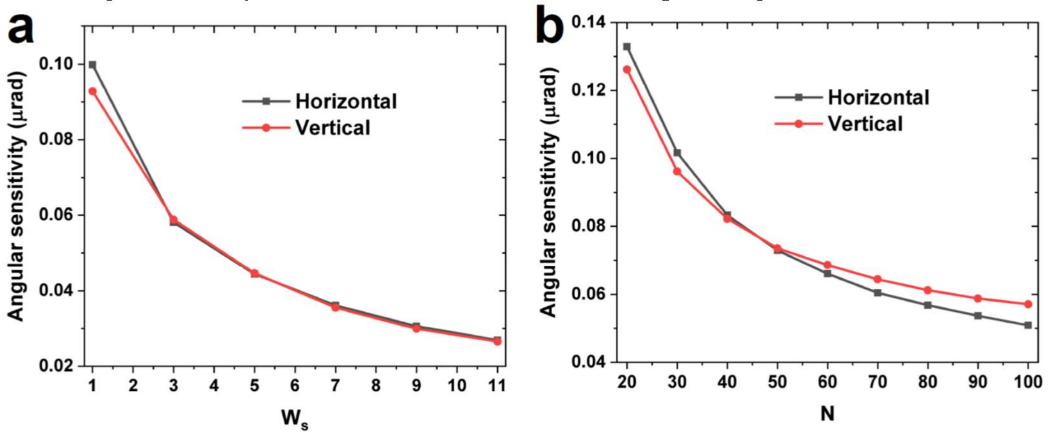

Fig. S2. (a) The angular sensitivity of the differential phase image changes (a) as function of window size W_S with speckle image number N=50, (b) as function of speckle image number N with window size $W_S = 3$.

In addition, the spatial correlation length will be affected by the window size. In order to improve the spatial resolution and reduce the correlation length, a smaller window size may be used at the cost of the angular sensitivity. Fig.S3 shows that the retrieved differential phase images of a sample star pattern change with different window size. For the smaller window size W_S =3 (a), the inner part of the star pattern can be clearly seen. In contrast, it is difficult to resolve the individual rays of the star near the center when the window size increases to W_S =11 (b).

As shown in Fig. S3, the sensitivity of the directional differential phase contrast is highly anisotropic. The bar pattern perpendicular to the gradient direction can be accessed from the phase gradient signal, while the one along the orthogonal direction remains undetected. The undetected bar pattern changes with the polar angle θ, and no single directional differential phase contrast image can provide the complete bar patterns. Therefore, it is essential to perform the omni-directional differential phase contrast imaging to depict the test sample in its entirety.

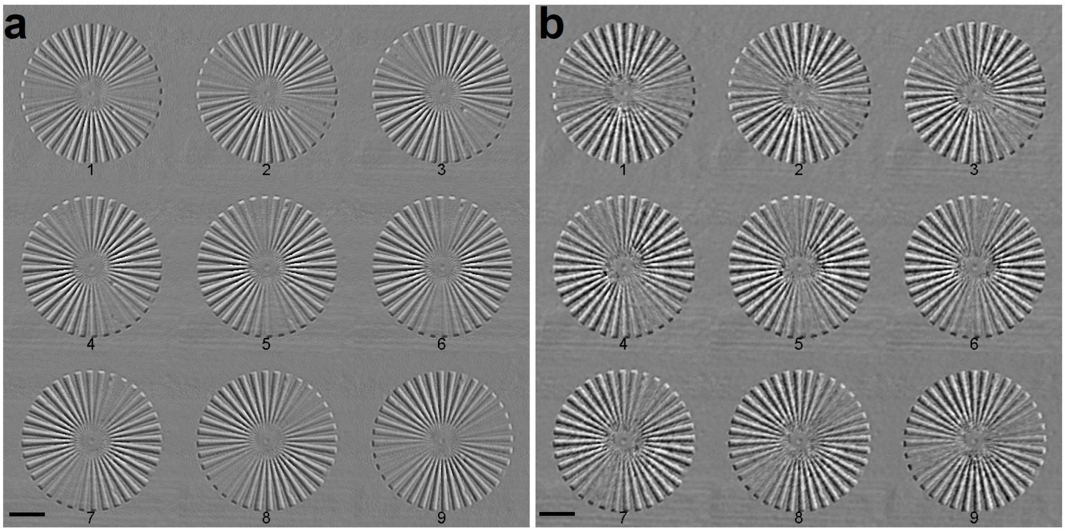

Fig. S3. The retrieved differential phase of a star pattern sample at different polar angle θ values (from (1) 0° to (9) 160° with 20° intervals) with window size (a) W_S =3 (b) W_S =11.The gray color for (a) and (b) indicates the gradient's variation from -1μrad (dark) to 1μrad (bright). The scale bar is 0.2mm.

References

1. Berujon S & Ziegler E (2016) X-ray Multimodal Tomography Using Speckle-Vector Tracking. *Physical Review Applied* 5(4):044014.
2. Zdora M-C*, et al.* (2017) X-ray Phase-Contrast Imaging and Metrology through Unified Modulated Pattern Analysis. *Phys. Rev. Lett.* 118(20):203903.
3. Wang H*, et al.* (2019) High-energy, high-resolution, fly-scan X-ray phase tomography. *Sci. Rep.* 9(1):8913.
4. Pfeiffer F*, et al.* (2007) High-resolution brain tumor visualization using three-dimensional x-ray phase contrast tomography. *Phys. Med. Biol.* 52(23):6923.
5. Diemoz PC, Bravin A, Langer M, & Coan P (2012) Analytical and experimental determination of signal-to-noise ratio and figure of merit in three phase-contrast imaging techniques. *Opt. Express* 20(25):27670-27690.
